# A simple and rapid protein purification method based on cell-surface display of SUMO-fused recombinant protein and Ulp1 protease

**DOI:** 10.1186/s13568-020-00999-4

**Published:** 2020-04-07

**Authors:** Xiao-Feng Zhou, Chen-Lu Zhang, Xue-Ping Gao, Wei-Long Wang, Zheng-Fen He, Feng-Ying Jiang, Yi-Lin Pang, Jiang-Hui Li, Xiao-Jun Ren, Huai-Bin Zhou, Guo-Qiang Tan, Jian-Xin Lyu, Wu Wang

**Affiliations:** 1 Zhejiang Provincial Key Laboratory for Technology and Application of Model Organisms, School of Laboratory Medicine and Life Sciences, Wenzhou Medical University, Wenzhou, 325035 Zhejiang China; 2 Department of Laboratory Medicine, Wuxi 9th People’s Hospital Affiliated to Soochow University, Wuxi, 214000 Jiangsu China

**Keywords:** Protein purification, Surface display, SUMO fusion, Ulp1

## Abstract

The development of novel methods for highly efficient protein purification remains a research focus in the biotechnology field because conventional purification approaches, including affinity purification, gel filtration, and ion-exchange chromatography, require complex manipulation steps and are costly. Here, we describe a simple and rapid protein purification strategy in which the SUMO tag and Ulp1 protease are surface-displayed separately on *Escherichia coli* cells. After protein induction, the cells are harvested, resuspended in cleavage buffer, and incubated together for cleavage. In this approach, the surface-displayed Ulp1 cleaves the membrane-anchored SUMO fusion protein, resulting in the release of the target protein from the C-terminal of SUMO into the solution. The bacterial cells harboring SUMO and Ulp1 on their surfaces can be easily removed by centrifugation. To evaluate the purification method, we used red fluorescent protein (mCherry). Purified mCherry protein (7.72 ± 1.05 mg from 1 L of bacterial culture) was obtained after only 30 min of incubation. The protein purity was higher than 80%, and could be further improved (> 90%) by simple ultrafiltration. This study offers a promising and simple strategy for the purification of recombinant protein in its native form that requires only cleavage and centrifugation steps.

## Introduction

Proteins are generally produced in heterologous systems because it is difficult to achieve satisfactory yields from natural sources. Efficient expression and purification of recombinant proteins remain fundamental issues for biotechnology. Various approaches have been used to simplify the purification process, such as the use of affinity (Kimple et al. 2013; Wood 2014) or self-aggregating tags (Lin et al. 2015a, b); however, these methods still have some drawbacks. For instance, while fusion with affinity tags has been considered the first-choice strategy because of its simplicity and high efficiency, the fused tag may alter the protein structure and thus affect protein function (Chen et al. 2015). Therefore, tag cleavage and removal are essential for the production of native proteins, which requires additional, time-consuming steps. Furthermore, the purification of intracellular recombinant proteins requires cell lysis. Recently, extracellular recombinant protein production has gained increasing attention owing to its advantages of avoiding the need for membrane disruption and enabling continuous production. Nevertheless, it still requires the recovery of the target proteins from the medium, which generally has a complex composition. In this study, to simplify protein purification and overcome the disadvantages of the above approaches, we developed a simple and rapid purification strategy based on the cleavage of a SUMO-fused recombinant protein by Ulp1 protease displayed on the surface of *Escherichia coli* (*E. coli*) cells.

**Fig. 1:**
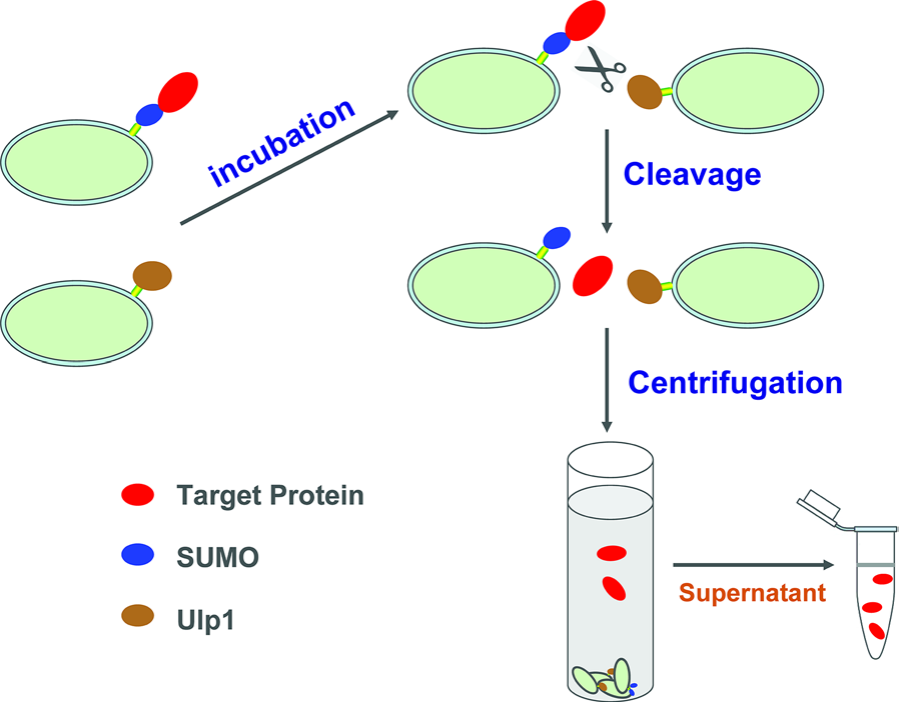
Illustration of the protein purification strategy based on cell-surface display of SUMO-fused recombinant protein and Ulp1 protease

SUMO is a ubiquitin-like protein (Johnson and Hochstrasser 1997) of approximately 11 kDa that is frequently used to improve target protein solubility and stability through N-terminal fusion (Bird 2011). The SUMO tag can be cleaved from the chimeric protein by Ulp1 protease, which specifically recognizes the SUMO tertiary structure, to generate the native protein without any redundant amino acids (Li et al. 2018). In light of the high efficiency of the SUMO-Ulp1 system in protein purification, we constructed two different vectors for the expression of SUMO-fused recombinant protein and Ulp1 protease on the surfaces of *E. coli* cells. The surface-displayed Ulp1 cleaves the N-terminal SUMO fusion protein, releasing the native target protein in the buffer solution. As SUMO and Ulp1 are anchored to the cell surface, they can be easily removed together with the bacterial cells by centrifugation. The feasibility of the simple and rapid protein purification approach that requires only cleavage and centrifugation steps was demonstrated. The overall strategy and manipulation steps for protein purification by this method are illustrated in Fig. 1.

## Materials and methods

### Vector construction

To construct a vector for the expression of surface-displayed SUMO-fused recombinant protein, an optimized DNA sequence encoding the N-terminal amino acids (1–29) of prolipoprotein (Lpp) (NP_416192), residues 45–159 of the outer-membrane protein OmpA (NP_415477) from *E. coli*, and SUMO (NP_010798.1) from *Saccharomyces cerevisiae* (*S. cerevisiae*), was designed considering the codon preference of *E. coli*, and synthesized at GenScript Biotech (Nanjing, China). The optimized *lpp*-*ompA*-*sumo* sequence (GenBank accession no. MT038411) was PCR-amplified using the primers Lpp-1/SUMO-2. To facilitate further DNA insertion, an NdeI recognition site (CATATG) was added to the 3′ terminus of *lpp*-*ompA*-*sumo*. Using an In-Fusion HD Cloning Kit (Clontech), the PCR product was subcloned into NcoI/XhoI-digested pET28b plasmid (Novogene) to generate the expression vector pET-Lpp-OmpA-SUMO (abbreviated as pET-LO-SUMO).

For the construction of a vector expressing surface-displayed Ulp1, an optimized DNA fragment encoding the N-terminal amino acids (1–28) of the autotransporter YfaL (NP_416736) from *E. coli* (Ko et al. 2012), residues 403–621 of Ulp1 (NP_015305) from *S. cerevisiae* (Lee et al. 2008) with a N-terminal His-tag, and the C-terminal amino acids (786–1250) of YfaL, was synthesized and PCR-amplified using the primers YfaL-Ulp1-1 and YfaL-Ulp1-2. The PCR product *yfal*-*ulp1* (GenBank accession no. MT179806) was ligated into pET28b at the NcoI and HindIII restriction sites, yielding the expression vector pET-YfaL-Ulp1.

A red fluorescent protein (mCherry) (Hui et al. 2018) with a C-terminal His-tag was used as a target protein to evaluate the feasibility of the purification strategy developed. The mCherry-coding gene sequence (GenBank accession no. MH070102) was synthesized and amplified by PCR using the primers mCherry-1 and mCherry-2. A vector for the expression of surface-displayed SUMO-fused mCherry, pET-LO-SUMO-mCherry, was constructed by inserting the *mcherry* DNA fragment into the NdeI restriction site of pET-LO-SUMO. A plasmid for the expression of intracellular mCherry with a C-terminal His-tag (pET-mCherry) was constructed in a similar manner, except that mCherry-3/mCherry-4 were used as primers and the NcoI/XhoI were chosen as the insertion sites in pET28b. All primers used are listed in Additional file 1: Table S1.

### Protein expression and identification

*Escherichia coli* BL21(DE3) cells containing pET-LO-SUMO-mCherry (pET- pET-LO-SUMO-mCherry/BL21) or pET-YfaL-Ulp1 (pET-YfaL-Ulp1/BL21) were grown in Luria–Bertani medium to an optical density at 600 nm (OD_600_) of 0.6–0.7. Then, protein expression was induced by the addition of 200 μM isopropyl-β-d-thiogalactoside (IPTG). After incubation at 16 °C for 24 h, the cells were harvested and resuspended in Tris buffer (20 mM Tris–HCl, pH 8.0) at an OD_600_ of 10. The whole-cell samples were mixed with 10 × loading buffer and heated at 100 °C for 10 min. The lysates were subjected to 12% SDS-PAGE. To verify the surface display of the proteins, cells were incubated with trypsin (200 μg/mL) at 37 °C for 1 h. The reaction was stopped by washing the cells twice with Tris buffer, and the recombinant proteins were detected again by SDS-PAGE using whole-cell lysates. For cell fractionation, IPTG-induced cells were harvested and resuspended in PBS buffer containing 1 mM EDTA and 10 μg/mL lysozyme at an OD_600_ of 5. After cell lysis by sonication, soluble cytoplasm and total cell membrane were separated by centrifugation (18,000 rpm, 1 h). The supernatant was regarded as the soluble cytoplasmic fraction. For further membrane fractionation, the pellet (total membrane fraction) was resuspended in PBS buffer containing 0.01 mM MgCl_2_ and 2% Triton X-100 to solubilize inner membrane materials and incubated at room temperature for 1 h. The outer membrane fraction was repelleted by ultracentrifugation (24,000 rpm, 1 h). The fractionated samples were stored until SDS-PAGE analysis as described above. For the immunolabeling of intact cells, IPTG-induced cells were incubated in anti-His antibody solution at 4 °C overnight and in secondary anti-mouse antibody solution for 2 h. After washing three times with Tris buffer, the cells were incubated in FAST ECL substrate solution (Thermo Pierce) for 10 min to develop the chemiluminescence. The luminescence intensity was measured at 425 nm using a microplate reader (MD SpectraMax M3).

### Cleavage of SUMO-fused mCherry by Ulp1 and purification of mCherry

IPTG-induced *E. coli* cells (pET-LO-SUMO-mCherry/BL21 and pET-YfaL-Ulp1/BL21) were washed twice by Tris buffer (20 mM Tris–HCl, pH 8.0) and resuspended in cleavage buffer (20 mM Tris–HCl, 150 mM NaCl, 2 mM dithiothreitol (DTT), pH 8.0) at an OD_600_ of 25. The bacterial suspensions were mixed together at a volume ratio of 1:1 and then incubated at 37 °C under mild shaking (100 rpm) for 30 min. Then, the sample was immediately centrifuged (12,000 rpm, 2 min), and the supernatant was transferred to new tube. The precipitate and supernatant were analyzed by SDS-PAGE and western blotting using anti-His antibody. Band intensity and protein purity on the SDS-PAGE gel or western blot film were analyzed using ImageJ software.

Several experimental conditions, including the ionic strength of the cleavage buffer and the incubation temperature and time, were tested to optimize the cleavage efficiency and target protein purity. The cells were resuspended in the cleavage buffer (20 mM Tris–HCl, 2 mM DTT, pH 8.0) containing different concentrations of NaCl (0, 150, and 500 mM). The cell suspension was incubated at 37 °C for 30 min before centrifugation. Following this, the cells in the cleavage buffer (20 mM Tris–HCl, 150 mM NaCl, 2 mM DTT, pH 8.0) were subjected to 30 min incubation at 4 °C, 16 °C, 30 °C, and 37 °C, respectively. Moreover, the cells in the same cleavage buffer were incubated at 37 °C for different incubation times (0, 5, 15, and 30 min). The supernatant of each sample was collected by centrifugation and analyzed using SDS-PAGE.

To further improve the purity of the target protein, the supernatant fraction was loaded into a 30-kDa ultrafiltration tube (Thermo Fisher), the eluate was collected after centrifugation (6000 rpm, 5 min), and its purity was evaluated using SDS-PAGE. The protein concentration of purified mCherry was measured at 280 nm using an extinction coefficient of 32.4 cm^−1^ mM^−1^.

## Results

### Construction of vectors for surface expression of SUMO-fused recombinant protein and Ulp1 protease on *E. coli* cells

**Fig. 2:**
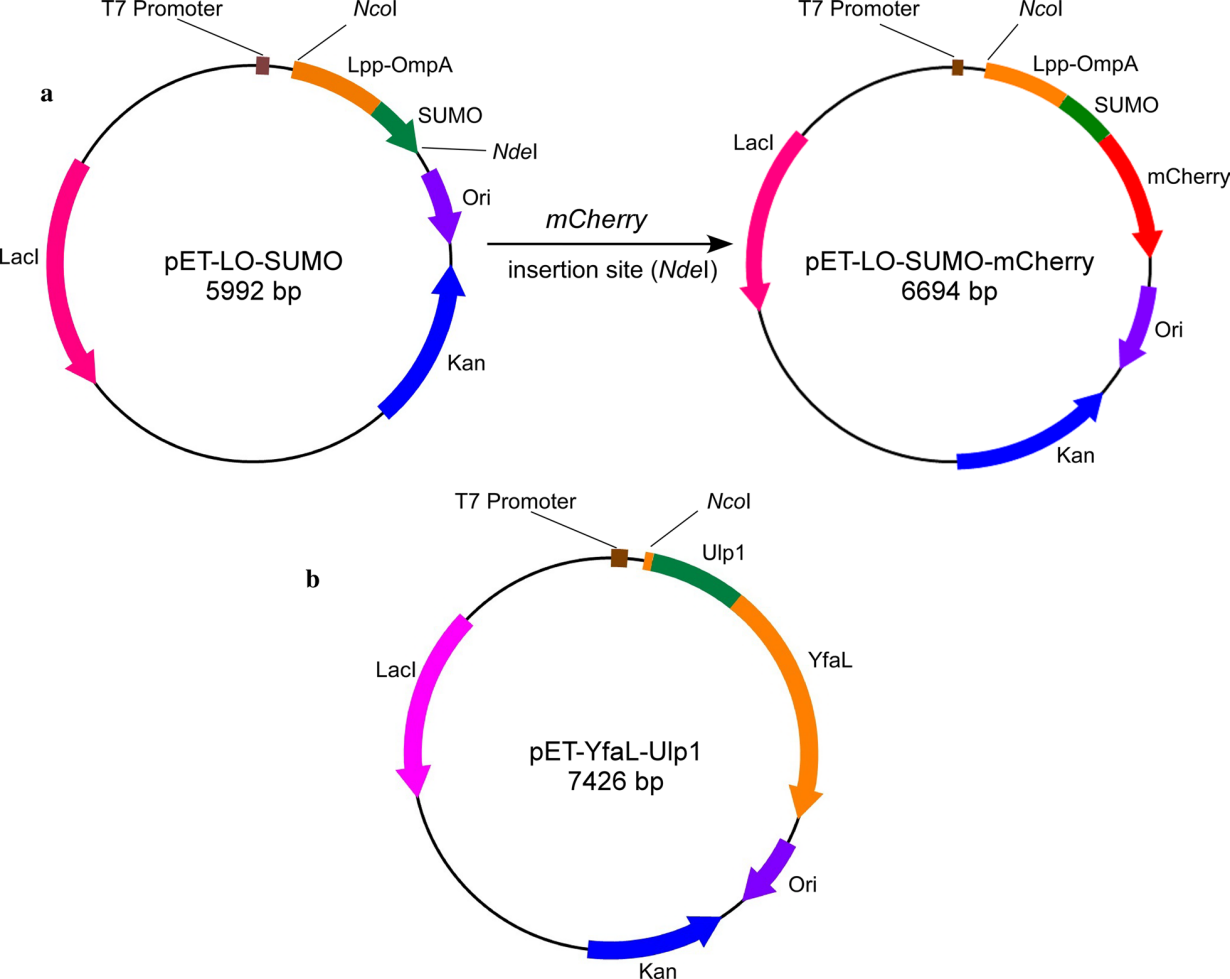
Schematic illustration of the vectors constructed for the expression of surface-displayed SUMO-fused recombinant protein (**a**) and Ulp1 protease (**b**). The coding sequence of the target protein can be inserted into the universal expression vector (**a**) at the NdeI restriction site

Various bacterial surface-display systems have been developed for wide applications (Schuurmann et al. 2014). In this study, SUMO was fused to the C-terminus of Lpp-OmpA for cell-surface expression of a SUMO-fused recombinant protein. To construct a universal vector for the expression of SUMO-fused recombinant protein, the NdeI recognition site was attached to the 3′ terminus of the *sumo* gene so that the recombinant protein-coding sequence can be easily subcloned into the vector at this restriction site. However, the expression of surface-displayed Ulp1 protease using the Lpp-OmpA carrier protein was unsuccessful (data not shown). Therefore, an autotransporter protein, YafL, was introduced to display the Ulp1 on the cell surface. The peptide fragment of YafL (29–785) was replaced with Ulp1 so that the C-terminal translocator (β-barrel) domain of YafL can deliver the passenger domain (Ulp1) across the outer membrane. Schematic illustrations of the plasmids expressing surface-displayed SUMO-fused recombinant protein (pET-LO-SUMO) and Ulp1 protease (pET-YfaL-Ulp1) are shown in Fig. 2a, b. mCherry was used as a target protein to demonstrate the feasibility of the innovative purification approach. A vector expressing surface-displayed mCherry (pET-LO-SUMO-mCherry) was constructed (Fig. 2a) and confirmed by direct sequencing, which confirmed that the 3′ terminus of *sumo* was immediately followed by the open reading frame of mCherry, without any gap.

### Surface expression and identification of SUMO-fused mCherry and Ulp1 on *E. coli* cells

**Fig. 3:**
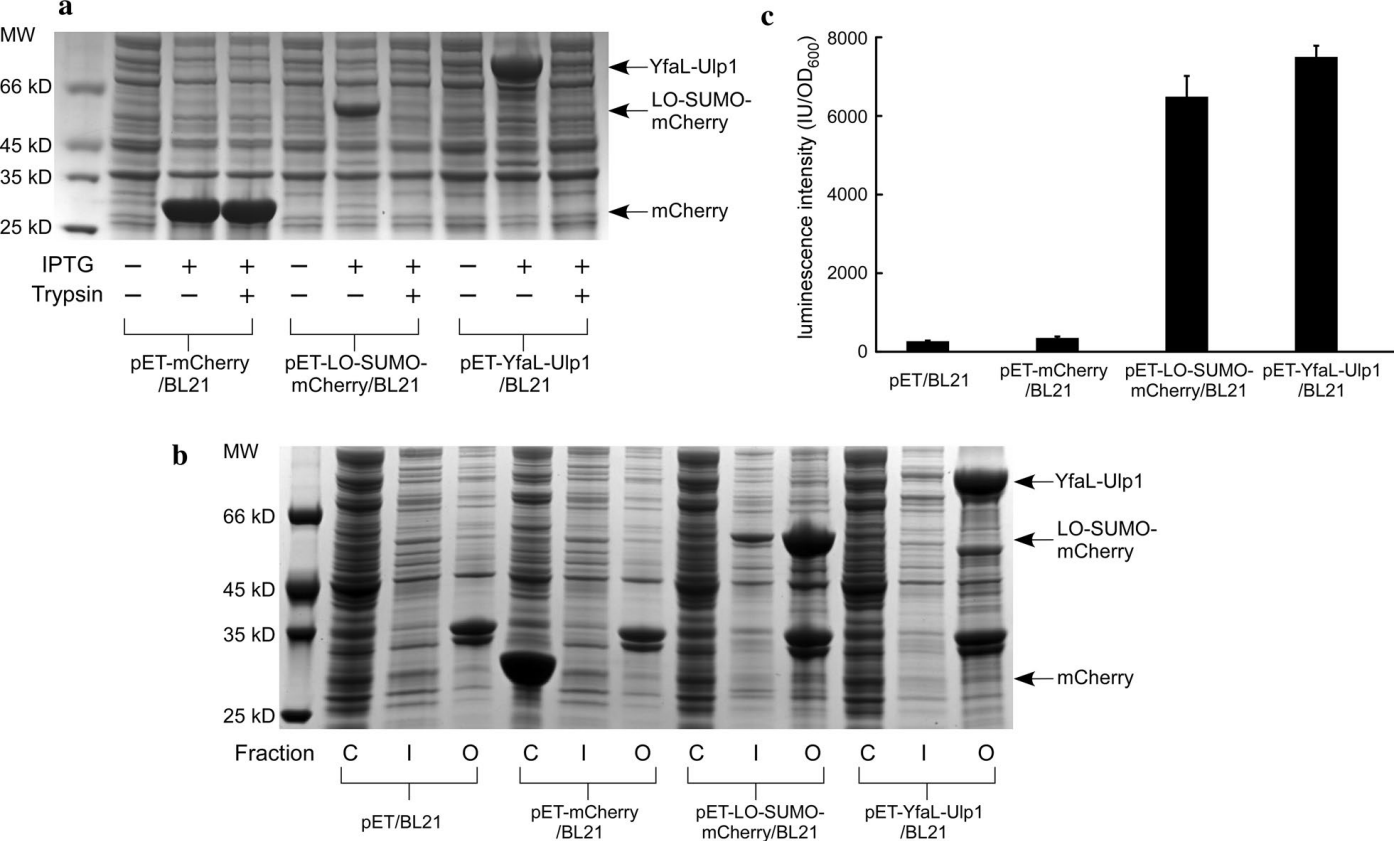
**a** SDS-PAGE analysis of the expression of surface-displayed SUMO-mCherry and Ulp1 induced by IPTG and the induced cells after incubation in the presence of 200 μg/mL trypsin at 37 °C for 1 h. **b** Protein expression in different fractions of induced cells. Equal volumes of fractionated samples were used for SDS-PAGE analyses. C: cytoplasmic fraction; I: inner membrane fraction; O: outer membrane fraction. The results are representative of three independent experiments. **c** Immunolabeling analysis of induced intact cells using anti-His antibody. After three washes to remove unbound secondary antibody, the cells were incubated in ECL substrate solution for 10 min to develop the chemiluminescence. The luminescence intensity was measured at 425 nm using a microplate reader. Data are means of three independent experiments, error bars represent standard deviation

We used the pET plasmid for protein expression because it contains the strong promoter, T7, which drives high protein expression in the presence of IPTG, resulting in high protein yield (Kaur and Kumar 2018). As can be seen in Fig. 3a, compared to samples without IPTG induction, LO-SUMO-mCherry and YfaL-Ulp1 were overexpressed after induction. The protein bands in the SDS-PAGE gel were consistent with the calculated molecular weights of the chimeric proteins (54 kDa and 80 kDa, respectively). To verify the cell surface localization of the two chimeric proteins, a protease accessibility experiment was conducted. As the protease cannot readily permeate through the cell membrane, intracellular proteins would remain intact, whereas those exposed on the cell surface would be digested. After 1 h of trypsin digestion, the surface-displayed chimeric proteins were completely degraded (Fig. 3a). Intracellular mCherry (pET-mCherry/BL21) included as a control was resistant to protease digestion under the same conditions (Fig. 3a). To directly identify the expression site of the two chimeric proteins in compartments of the recombinant cells, a cell fractionation experiment was performed. As shown in Fig. 3b, the majority of both proteins (LO-SUMO-mCherry and YfaL-Ulp1) were detected in the outer membrane fraction, whereas mCherry from control cells (cytosolic expression) was found in the soluble cytoplasmic fraction. To confirm the surface display, an anti-His antibody was exogenously added to intact cells. Because antibodies cannot diffuse through the cell membrane, the location of fusion proteins on the cell surface can be revealed in this manner. Very high luminescence intensity was detected on cells expressing LO-SUMO-mCherry or YfaL-Ulp1 (Fig. 3c). In contrast, only weak luminescence was observed in the cytosol-expressing strain or the negative control strain (pET/BL21), probably due to nonspecific binding of the anti-His antibody to the cell surface (Fig. 3c). The above results collectively suggested that SUMO-fused mCherry and Ulp1 were effectively displayed on the cell surface.

### Cleavage of surface-displayed SUMO-fused mCherry by surface-displayed Ulp1 and purification of mCherry

**Fig. 4:**
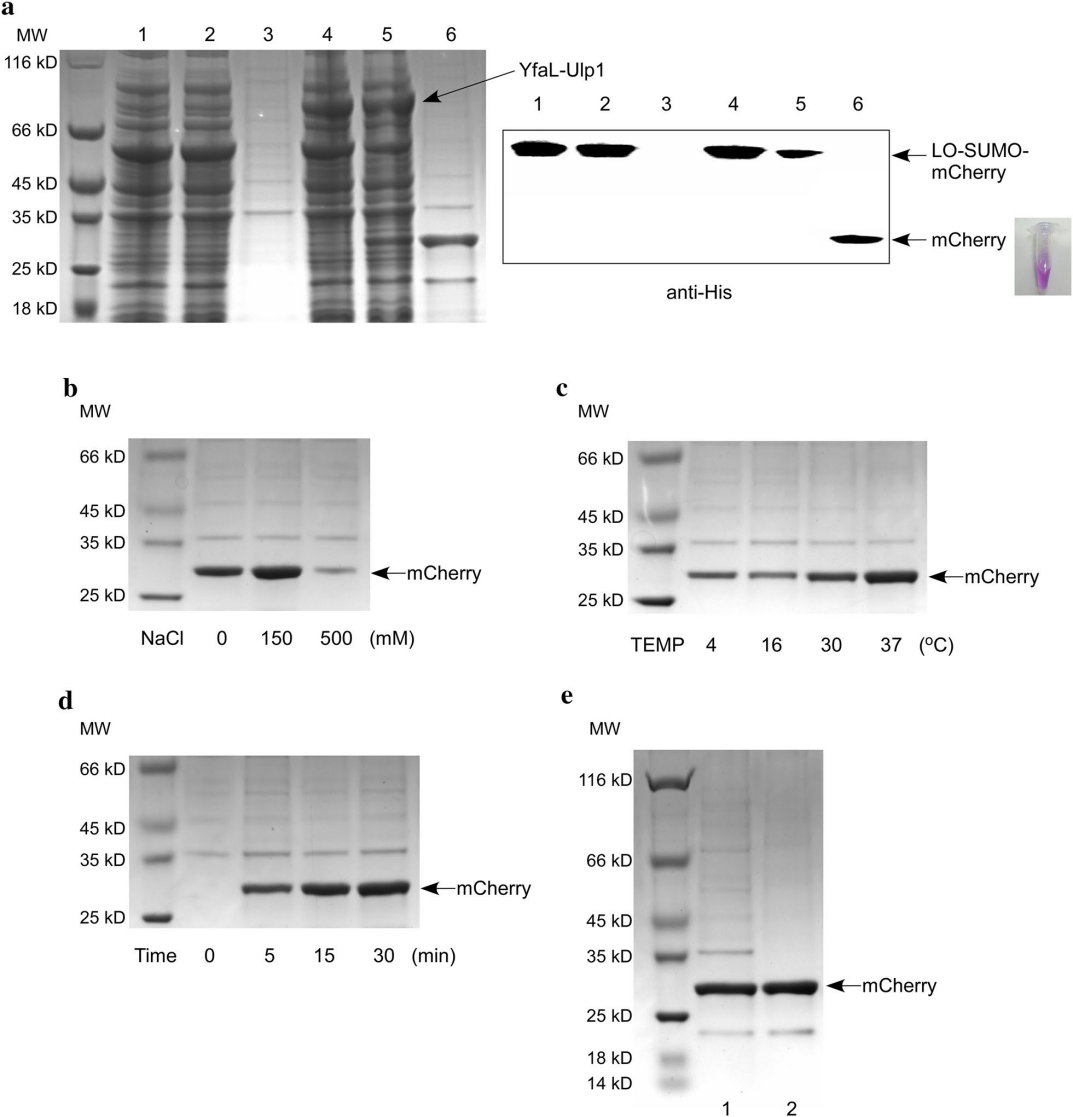
Cleavage of surface-expressed SUMO-fused mCherry by surface-expressed Ulp1 and purification of mCherry. **a** SDS-PAGE analysis (left panel) and western blot (right panel) of surface-displaying bacterial cells and released target protein fractions. IPTG-induced *E. coli* cells (pET-LO-SUMO-mCherry/BL21 and pET-YfaL-Ulp1/BL21) were incubated together in cleavage buffer at 37 °C for 30 min, after which the cells and the buffer solution were immediately separated by centrifugation. Lane 4: cell pellets before incubation; lane 5: cell pellets after incubation; lane 6: supernatant fraction after incubation. The negative control (pET-LO-SUMO-mCherry/BL21 and pET/BL21) was prepared using the same procedures (Lane 1–3). Cleavage was conducted under different conditions of ionic strength (**b**), temperature (**c**), and incubation time (**d**) to optimize the cleavage efficiency and mCherry protein yield. The supernatant fraction after cleavage and centrifugation was analyzed by SDS-PAGE. **e** SDS-PAGE analysis of purified mCherry protein prior to (lane 1) or after (lane 2) ultrafiltration using a 30-kDa ultrafiltration tube. The results are representative of three independent experiments

Protein recovery and subsequent concentration are necessary for the extracellular protein production and purification, because the recombinant protein is secreted and diluted into the culture medium. To avoid this, in our study, the induced *E. coli* cells were harvested and resuspended in cleavage buffer at a high density (OD_600_ = 25). Then, the IPTG-induced cells (pET-LO-SUMO-mCherry/BL21 and pET-YfaL-Ulp1/BL21) were mixed and incubated at 37 °C for 30 min. Surface cleavage of SUMO-fused mCherry by Ulp1 protease was evaluated by SDS-PAGE. As shown in Fig. 4a, the protein band of approximately 54 kDa, which corresponds to uncleaved LO-SUMO-mCherry, was much less intense after cleavage than before. In the pelleted cell fraction, a band of 27 kDa, corresponding to the LO-SUMO chimeric fragment, emerged. Interestingly, the supernatant had a red color, suggesting that the protease cleavage had successfully released the mCherry protein from the chimeric protein into the buffer. In accordance herewith, SDS-PAGE analysis revealed a band of 27 kDa, corresponding to the mCherry protein, in the supernatant fraction, and its purity was higher than 80%. When mCherry surface-displayed cells were incubated with cells lacking the protease (pET/BL21) under the same experimental conditions, the protein band of LO-SUMO-mCherry remained intact, and no mCherry protein was detected in the supernatant. This finding suggested that Ulp1 is indispensable for the cleavage of SUMO-fused protein on the cell surface. Because LO-SUMO and mCherry have similar molecular weights, they cannot be easily differentiated by SDS-PAGE. Therefore, we used a His-tagged mCherry and verified cleavage by western blotting using an anti-His antibody. The western blot results were in agreement with those of SDS-PAGE and revealed that the cleavage efficiency of SUMO-fused mCherry by Ulp1 displayed on the *E. coli* cell surface was approximately 60% (Fig. 4a).

Next, we optimized experimental conditions, including the ionic strength of the cleavage buffer and the incubation temperature and time, to improve the cleavage efficiency and target protein purity. As shown in Fig. 4b, compared to cleavage buffer without any salt, a low concentration of NaCl (150 mM) improved protein yield, whereas high ionic strength (500 mM NaCl) dramatically inhibited cleavage. The cleavage activity of Ulp1 was the highest at 37 °C (Fig. 4c). As expected, the amount of mCherry released in the supernatant fraction increased in a time-dependent manner over the 30-min cleavage incubation (Fig. 4d). When the incubation period was prolonged, the protein yield did not significantly increase, and many other proteins were secreted into the supernatant (results not shown). Based on these findings, a 30-min incubation at 37 °C in the defined cleavage buffer was considered an optimal condition for protein purification.

As shown in Fig. 4a (lane 6), several impurities were detected in the supernatant after cleavage under the optimized conditions, although the bands corresponding to these proteins were very faint. To improve the purity of the target protein further, a simple ultrafiltration (using a 30-kDa ultrafiltration tube) was applied, because most of the impurities were larger than 30 kDa. The eluate after centrifugation was collected. As shown in Fig. 4e, the majority of the impurities disappeared after ultrafiltration, and the purity of the mCherry protein was higher than 90%. The final yield of purified mCherry obtained from 1 L of bacterial culture was 7.72 ± 1.05 mg.

## Discussion

Cell-surface display entails the display of a target protein on the surfaces of microbial cells through fusion with an anchoring motif, and has been a useful approach for various purposes, such as the development of live vaccines, whole-cell biocatalysts, and environmental bio-adsorbents, and the screening of protein libraries (Lee et al. 2003). Surface-anchoring motifs, including OmpA, ice nucleation protein (INP) (Li et al. 2004), and auto-transporters (van Ulsen et al. 2018), act as carriers to deliver the target proteins across the bacterial cell membrane. Among them, Lpp-OmpA was frequently used (Fasehee et al. 2018; Wang et al. 2019; Wei et al. 2014) and was considered an ideal carrier protein (Lang 2000). For example, *E. coli* cells expressing Lpp-OmpA-anchored EC20 (a metal-binding protein) accumulated substantially more mercury than did cells engineered with INP-fused EC20 (Bae et al. 2002). However, the Lpp-OmpA system seems not applicable to all proteins. In this study, the Ulp1 protease could not be surface-expressed using the Lpp-OmpA system; however, after fusion with YfaL, it was effectively surface-displayed, implying that cell-surface display is influenced by various factors, such as the protein structure or molecular size. This also indicates that such factors should be considered when selecting anchoring proteins and that surface-display systems can be optimized by using different anchoring proteins.

Protein purification is costly and laborious, especially at the industrial scale. To shorten the processing time, extracellular recombinant protein expression is considered a useful strategy as the cell disruption step can be omitted (Burdette et al. 2018). Although this approach simplifies the purification process, the protein still needs to be separated from the culture medium. Recently, a new purification method was developed, in which the sortase recognition site (LPQTG) was inserted between the Lpp-OmpA anchor and the recombinant protein on the cell surface, to allow cleavage of the target protein from the Lpp-OmpA carrier by sortase treatment (Fasehee et al. 2018). However, the sortase was used in the soluble form and thus, further purification was required to separate it from the target protein, which was also present in the soluble form in the supernatant. In addition, sortase cleaves between the threonine and glycine residues of the LPXTG motif and therefore, the obtained protein is not native, but carries an extra glycine residue at the N-terminus. In another new strategy, the antimicrobial peptides were secreted and formed extracellular amyloid aggregates on the *E. coli* cell surface (Wang et al. 2017). The surface-displayed peptide was fused to a self-cleaving intein (*Mxe* GyrA) and released through DTT treatment. Although no protease is required in this self-cleaving approach, it takes a long time (at least 12 h) to achieve a considerable protein yield, and because of the continuous secretion of other host proteins, this will decrease protein purity. Another drawback of this approach is that the N-terminal signal peptide cannot be removed from the final purified protein. In this work, we developed a novel protein expression and purification strategy in which the SUMO tag and Ulp1 protease are surface-displayed separately on *E. coli* cells. The cells harboring the surface-immobilized SUMO and Ulp1 can be easily removed by simple centrifugation, with the purified protein being contained in the supernatant.

Because of the high protease activity of Ulp1 towards SUMO fusion, a high protein yield of mCherry (7.72 mg/L) can be achieved within half an hour by this method, which is considerably higher than yields achieved with previously reported approaches (Sevastsyanovich et al. 2012; Sichwart et al. 2015) for production of the same protein through extracellular secretion and accumulation. The low temperature (16 °C) used for protein induction in this study probably also contributed to the enhanced protein yield of mCherry, as expression at a low temperature is a well-known technique to avoid or limit aggregation of the recombinant protein and yield more soluble and well-folded protein (Kaur and Kumar 2018). However, because of the limited surface area, the yield of surface-displayed mCherry was much lower than that of protein expressed in the cytoplasm, which was 28.6 mg/L when purified from pET-mCherry/BL21 by affinity chromatography using a Ni-agarose column. Therefore, a novel strategy to further increase the yield of cell-surface displayed protein is required.

It should be noted that the protein purity achieved in the present study was still insufficient, although most of the impurities could be removed after passage through a 30-kDa ultrafiltration tube. Therefore, further research efforts are needed to improve protein purity in several aspects. As intracellular proteins are transported across the cell membrane mostly via bacterial carrier proteins (Thanassi and Hultgren 2000), deletion of these proteins by genetic engineering would reduce non-target protein secretion. Moreover, chemicals that can inhibit protein secretion might also be useful to improve protein purity. Although further studies are needed before this protein purification strategy can be widely applied, our present work provides a promising approach for highly efficient protein purification.

## Supplementary information

**Additional file 1: Table S1.** Primers used for vector construction.

## Data Availability

The optimized gene sequences for expression of surface-displayed SUMO fusion protein (*lpp*-*ompA*-*sumo*) and Ulp1 (*yfal*-*ulp1*) have been submitted to NCBI Genbank database with the accession number MT038411 and MT179806.

## Abbreviations

DTT: Dithiothreitol
IPTG: Isopropyl-β-d-thiogalactoside
OD: Optical density
SDS-PAGE: Sodium dodecyl sulfate-polyacrylamide gel electrophoresis
EDTA: Ethylenediaminetetraacetic acid
