# Supplementary material for: A simple and rapid protein purification method based on cell-surface display of SUMO-fused recombinant protein and Ulp1 protease
Source: AMB Express. 2020 Apr 7;10:65. doi: 10.1186/s13568-020-00999-4 (PMC7138890; doi:10.1186/s13568-020-00999-4)
Supplement: Supplementary file 1 — Additional file 1: Table S1. Primers used for vector construction. [file 13568_2020_999_MOESM1_ESM.doc]

**Table S1** Primers used for vector construction

| Primer name | Primer Sequence（5′→3′） |
| --- | --- |
| Lpp-1 | CTTTAAGAAGGAGATATACCATGAAGGCGACCAAACTGGTGCTGG |
| SUMO-2 | GGTGGTGGTGGTGGTGCTCGAGCATATGACCTCCAATCTGTTCGCGGTGAGC |
| YfaL-Ulp1-1 | CTTTAAGAAGGAGATATACCATGCGTATCATTTTCCTGCGTAAGGAG |
| YfaL-Ulp1-2 | TGCTCGAGTGCGGCCGCAAGCTTTTACCATTTCACGGTCATGCTCAGAAAAC |
| mCherry-1 | CCGCGAACAGATTGGAGGTATGGTCTCTAAAGGCGAGGAAG |
| mCherry-2 | GGTGGTGGTGGTGCTCGAGTTTGTACAGTTCGTCCATGCCG |
| mCherry-3 | CTTTAAGAAGGAGATATACCATGGTCTCTAAAGGCGAGGAAG |
| mCherry-4 | GGTGGTGGTGGTGGTGCTCGAGTTTGTACAGTTCGTCCATGCCG |
